# Modeling the natural history of ductal carcinoma in situ based on population data

**DOI:** 10.1186/s13058-020-01287-6

**Published:** 2020-05-27

**Authors:** Sarocha Chootipongchaivat, Nicolien T. van Ravesteyn, Xiaoxue Li, Hui Huang, Harald Weedon-Fekjær, Marc D. Ryser, Donald L. Weaver, Elizabeth S. Burnside, Brandy M. Heckman-Stoddard, Harry J. de Koning, Sandra J. Lee

**Affiliations:** 1 Department of Public Health, Erasmus MC, University Medical Center Rotterdam, P.O. Box 2040, 3000 CA Rotterdam, Netherlands; 2 Department of Data Sciences, Dana-Farber Cancer Institute, Boston, MA USA; 3 Department of Biostatistics, Harvard T.H. Chan School of Public Health, Boston, MA USA; 4 Oslo Center for Biostatistics and Epidemiology, Research Support Services, Oslo University Hospital, Oslo, Norway; 5 Department of Population Health Sciences, Duke University Medical Center, Durham, NC USA; 6 Department of Mathematics, Duke University, Durham, NC USA; 7 Department of Pathology and Laboratory Medicine, Larner College of Medicine, University of Vermont and UVM Cancer Center, Burlington, VT USA; 8 Radiology Department, University of Wisconsin School of Medicine and Public Health, Madison, WI USA; 9 Division of Cancer Prevention, National Cancer Institute, Bethesda, MD USA

**Keywords:** United States, Breast neoplasms, Early detection of cancer, Disease progression, Breast carcinoma in situ

## Abstract

**Background:**

The incidence of ductal carcinoma in situ (DCIS) has increased substantially since the introduction of mammography screening. Nevertheless, little is known about the natural history of preclinical DCIS in the absence of biopsy or complete excision.

**Methods:**

Two well-established population models evaluated six possible DCIS natural history submodels. The submodels assumed 30%, 50%, or 80% of breast lesions progress from undetectable DCIS to preclinical screen-detectable DCIS; each model additionally allowed or prohibited DCIS regression. Preclinical screen-detectable DCIS could also progress to clinical DCIS or invasive breast cancer (IBC). Applying US population screening dissemination patterns, the models projected age-specific DCIS and IBC incidence that were compared to Surveillance, Epidemiology, and End Results data. Models estimated mean sojourn time (MST) in the preclinical screen-detectable DCIS state, overdiagnosis, and the risk of progression from preclinical screen-detectable DCIS.

**Results:**

Without biopsy and surgical excision, the majority of DCIS (64–100%) in the preclinical screen-detectable state progressed to IBC in submodels assuming no DCIS regression (36–100% in submodels allowing for DCIS regression). DCIS overdiagnosis differed substantially between models and submodels, 3.1–65.8%. IBC overdiagnosis ranged 1.3–2.4%. Submodels assuming DCIS regression resulted in a higher DCIS overdiagnosis than submodels without DCIS regression. MST for progressive DCIS varied between 0.2 and 2.5 years.

**Conclusions:**

Our findings suggest that the majority of screen-detectable but unbiopsied preclinical DCIS lesions progress to IBC and that the MST is relatively short. Nevertheless, due to the heterogeneity of DCIS, more research is needed to understand the progression of DCIS by grades and molecular subtypes.

## Introduction

With the introduction of mammographic screening, the incidence of ductal carcinoma in situ (DCIS) has increased rapidly due to the ability of mammography to identify associated microcalcifications. In the absence of calcifications that are observable by mammography, DCIS is either undetectable or preclinical, detected incidentally during biopsy of a different lesion, or rarely, detected clinically when it induces fibrosis and produces a clinical mass. In the USA, DCIS incidence rate among women older than 40 years increased from 5.6 per 100,000 women in 1990–1994 to 31.6 per 100,000 women in 2010–2014 [1]. DCIS is regarded as “a neoplastic proliferation of cells within the ductal-lobular structures of the breast that has not penetrated the myoepithelial-basement membrane interface” [2]. Although DCIS itself is not life-threatening, it can progress to invasive breast cancer (IBC) if left untreated [3, 4]. The most common treatment options for DCIS are breast-conserving surgery, usually with breast irradiation, and total mastectomy [5]. Although the detection of DCIS has increased substantially relative to detection of invasive breast cancer, its natural history remains poorly understood. In particular, there is considerable uncertainty about the rates of DCIS progression and regression.

To date, two approaches have been used to characterize DCIS natural history. In the first approach, observational studies have focused on women with a biopsy-confirmed diagnosis of DCIS who did not undergo definitive surgery [3, 4, 6–12]. However, because such observational studies report on outcomes in patients who received core needle or excisional biopsies, the findings do not directly ascertain the unobserved natural history of progression. To address this caveat, a second approach uses mathematical models in conjunction with clinical data and/or data from mammography screening studies to infer latent disease dynamics [13–16]. Estimates of progression risk and mean sojourn time (MST), that is, the time from preclinical DCIS to IBC, vary widely between the two approaches and even between studies of the same approach. For instance, progression risk estimates are generally lower for biopsy-treated women in observational studies (ranging from 12 to 52% [4, 6–12]) than from modeling studies (ranging from 61 to 91% [13, 14, 17, 18]).

Due to the residual uncertainty about natural history, the extent of breast cancer overdiagnosis and overtreatment are difficult to quantify. Indeed, while researchers generally accept that a fraction of screen-detected DCIS and IBC lesions would not progress to clinical disease if left untreated [10], estimates of overdiagnosis range from less than 1% [19] to 37% [13]. Currently ongoing active monitoring trials for low-risk DCIS patients [20–22] are expected to provide an estimate of the risk of progression from biopsy-confirmed DCIS to invasive disease. However, these trials rely on statistical inference rather than direct observation to provide insight into unobservable natural history dynamics.

Here we developed an alternative modeling approach to study DCIS natural history. Rather than relying on data from screening studies, we employed population-based models of incidence and progression in conjunction with breast cancer incidence data from the Surveillance, Epidemiology and End Results (SEER) program. By comparing two independently developed and validated population models of breast cancer, and evaluating multiple submodels for each, we explored a range of possible natural histories and projected the resulting extent of overdiagnosis.

## Materials and methods

### Model description

Two well-established models have been used for this study, which were developed in the Cancer Intervention and Surveillance Modeling Network (CISNET): model D (Dana-Farber Cancer Institute) [23, 24] and model E (Erasmus MC, University Medical Center Rotterdam) [25, 26].

Model D is an analytical model that estimates breast cancer incidence and mortality as a function of disease natural history, detection process, and treatment [23, 27]. Details of IBC [24, 28] and DCIS [23, 29] modeling have been reported previously. DCIS natural history parameters were estimated using data from the Norwegian breast cancer screening program. Details of model D and estimation of model parameters can be found in Additional file 1.

Model E is a discrete event-driven micro-simulation model that simulates independent life histories of women using a parallel universe approach [25, 26]. This means that there are two identical female populations whereby one population undergoes screening and one population does not have screening. Recently, model E has been extended by including DCIS [30]. The DCIS model component is stage-specific whereby preclinical undetectable DCIS can be allowed to enter a regression state or progress to preclinical screen-detectable DCIS, clinical DCIS, or preclinical IBC. The natural history parameters for DCIS were estimated through calibration with DCIS SEER data. A more extensive description of model E can be found in Additional file 2.

Both models have been extensively evaluated on US breast cancer incidence and mortality trends [31–33] and used to estimate benefits and harms of mammography screening [34]. Both models have been used to inform breast cancer screening recommendations, for instance, by the US Preventive Services Task Force (USPSTF) [35]. A more detailed description of models D and E can be found on https://resources.cisnet.cancer.gov/registry/packages/filter/Breast/, in the Additional files and elsewhere [23, 34].

### Submodels

**Fig. 1:**
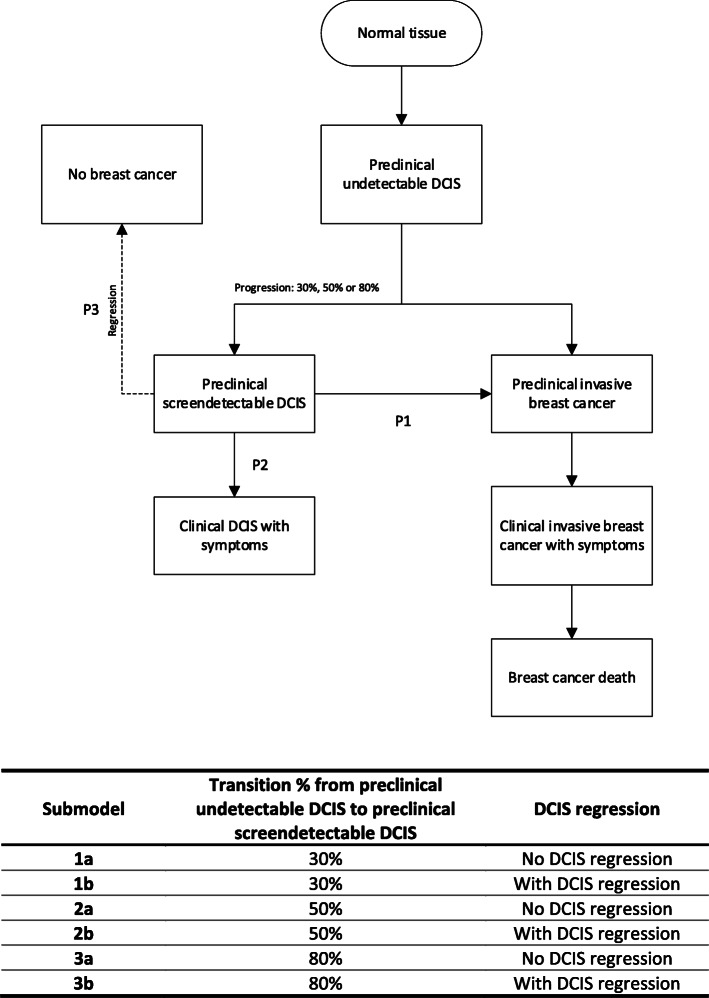
Graphical representation of the DCIS model and its submodels. The figure depicts the states that are included in the model. The dotted arrow from the state preclinical screen-detectable DCIS to the state no breast cancer is included into the submodels where DCIS regression is allowed. The proportion of preclinical undetectable DCIS that progresses to preclinical screen-detectable DCIS can be 30%, 50%, or 80%. Different model assumptions on the natural history of DCIS are included for each submodel

In total, 6 parallel submodels were developed (represented in Fig. 1), each with its own assumptions common for both model D and model E. This includes three different proportions of DCIS lesions in the preclinical undetectable DCIS state progressing to preclinical screen-detectable DCIS (i.e., 30%, 50%, and 80%), and the ability of DCIS to regress [23, 25]. The structure of the DCIS components in models D and E is similar as they both assume that normal breast tissue can progress to preclinical DCIS which is undetectable as depicted in Fig. 1. From there onwards, preclinical undetectable DCIS can become screen-detectable or evolve into preclinical IBC. Screen-detectable DCIS can progress to preclinical IBC (P1) or clinical DCIS (P2), whereas preclinical IBC can develop into clinical IBC possibly leading to breast cancer death. The death state unrelated to breast cancer can result from any non-breast cancer death state in Fig. 1.

### Input parameters

Estimated from Breast Cancer Surveillance Consortium (BCSC) data, mammography sensitivity for detecting DCIS were set to 0.45, 0.55, 0.70, and 0.85 for the periods 1975–1984, 1985–1999, 2000–2009, and 2010 onwards, respectively [24]. This reflects the improvement in mammography sensitivity over time in the USA [36]. Furthermore, both models used screening dissemination patterns in the USA over time until 2010 and extrapolated patterns thereafter [37]. Other common input parameters, such as birth tables, life tables, and age-period-cohort estimates of breast cancer incidence in the absence of screening, are described in a previous publication [37].

### Model outcomes

The models estimated rates of overdiagnosis of DCIS and IBC (per 100,000 women) and MSTs in the screen-detectable preclinical DCIS state. DCIS overdiagnosis was defined as a screen-detected DCIS case that would not have been found in the absence of screening as it would not have progressed to clinical DCIS or clinical IBC in the remaining lifetime of the woman. Similarly, overdiagnosed IBC was defined as a screen-detected IBC case that would not have been found in the absence of screening as it would not have developed into clinical IBC in the remaining lifetime of the woman.

We also calculated overdiagnosis for DCIS, IBC, and DCIS and IBC combined by dividing the number of overdiagnosed cases by the total number of screen-detected and clinically detected cases. The MST was defined as the number of years in which DCIS is screen-detectable before it progresses to another state, which could be the preclinical IBC state, clinical DCIS state, or normal tissue (DCIS regression). For model D, the MST was an input parameter (Additional file 1) while it was an output for model E.

### Statistical analysis

We generated age-specific and age-adjusted (between ages 30 and 79 years) data for birth cohorts born between 1890 and 1996. The projected age-adjusted incidence for DCIS and IBC were compared with SEER data. A chi-square test was performed to assess the goodness of fit between the projected incidence estimates and observed data of DCIS and IBC for the calendar year 1975 to 2015.

## Results

### Comparing SEER data and projected incidence for DCIS and IBC

**Fig. 2:**
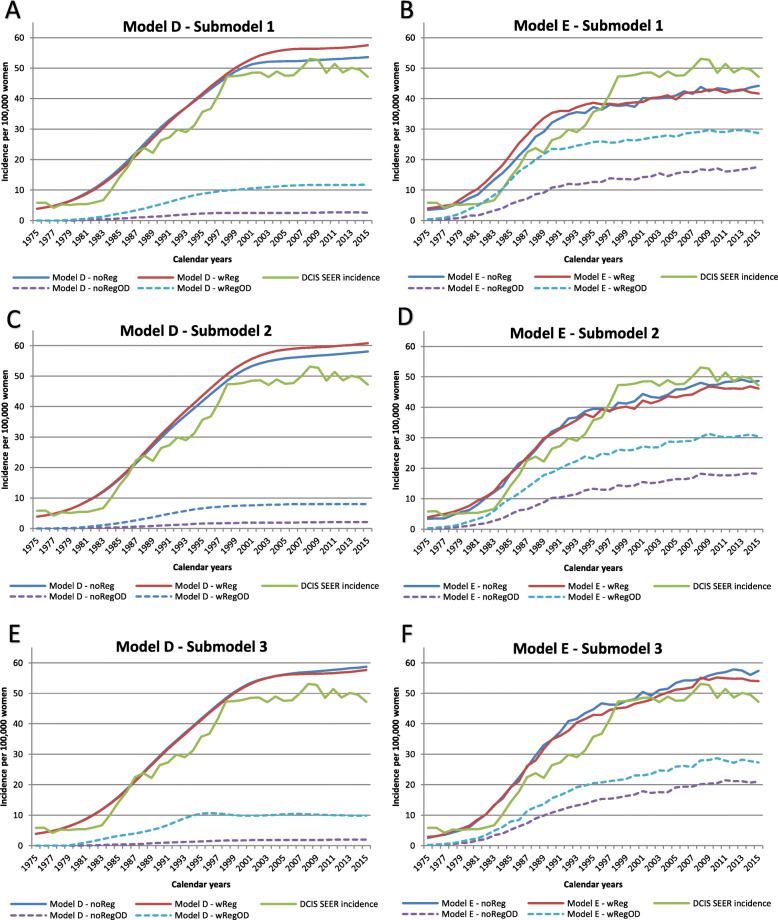
DCIS incidence and overdiagnosis. Each graph includes SEER data and 2 submodels (1 without DCIS regression and 1 with DCIS regression). The projections include women in the age group 30–79 years. noReg, model without DCIS regression; wReg, model with DCIS regression; OD, overdiagnosis

**Fig. 3:**
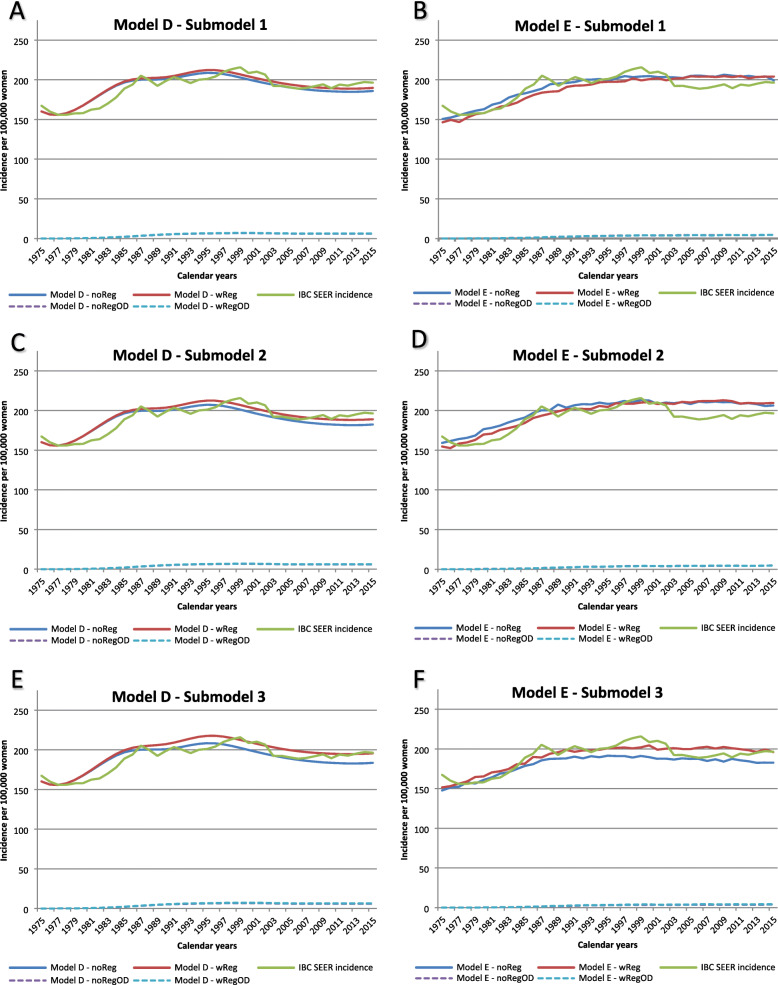
IBC incidence and overdiagnosis. Each graph includes SEER data and 2 submodels (1 without DCIS regression and 1 with DCIS regression). The projections include women in the age group 30–79 years. noReg, model without DCIS regression; wReg, model with DCIS regression; OD, overdiagnosis

Model D projected DCIS incidence that matched the SEER data relatively well for all submodels as indicated by the goodness-of-fit measures (Table 1). The model fit was better for earlier calendar years (Fig. 2). Model D showed higher DCIS incidence in later years when regression was allowed compared to no regression, except for submodel 3 where the incidence was similar regardless of DCIS regression. Model E also generated DCIS incidence that matched the SEER data relatively well as shown in Table 1, except for submodel 1b. The best fit for model E was submodels 2a and 2b where 50% of the DCIS lesions in the preclinical undetectable DCIS state progress to preclinical screen-detectable DCIS state. Model E had lower DCIS incidences when regression was allowed in submodels 2 and 3 but not 1. IBC incidence projected from both models D and E matched the SEER data relatively well for all submodels (Fig. 3).

**Table 1:** Outcomes for three DCIS submodels with and without regression during 1975–2015 for women aged 30–79 years

		Df	1a noReg	1b wReg	2a noReg	2b wReg	3a noReg	3b wReg
Goodness-of-fit Deviance (observed-estimated)^2								
DCIS deviance (*p* value*)	Model D	40	665 (0.98)	1182 (0.82)	1242 (0.79)	2168 (0.15)	1307 (0.77)	1121 (0.89)
	Model E	40	1505 (0.18)	2071 (0.004)	698 (0.95)	926 (0.81)	1637 (0.12)	1064 (0.56)
IBC deviance (*p* value*)	Model D	40	2734 (1.00)	2499 (1.00)	3406 (1.00)	2585 (1.00)	3116 (1.00)	3527 (1.00)
	Model E	40	3183 (1.00)	4386 (0.98)	6071 (0.86)	5388 (0.95)	5921 (0.80)	2510 (1.00)
Mean sojourn time								
MST of preclinical screen-detectable DCIS before progressing to preclinical IBC	Model D		2.5	1.5	0.7	0.5	0.4	0.2
	Model E		0.9	0.5	0.8	0.4	1.0	0.6
MST of preclinical screen-detectable DCIS before progressing to clinical DCIS	Model D		2.5	1.5	1.9	1.9	1.9	1.9
	Model E		3.9	3.1	4.5	0.9	7.7	1.4
MST of preclinical screen-detectable DCIS before regressing	Model D		NA	1.5	NA	1.5	NA	1.5
	Model E		NA	4.0	NA	1.3	NA	0.6
Overdiagnosis								
% DCIS overdiagnosis	Model D		4.8%	19.3%	3.4%	13.3%	3.1%	19.1%
	Model E		35.2%	65.8%	33.7%	62.2%	33.6%	47.8%
% IBC overdiagnosis	Model D		2.4%	2.4%	2.4%	2.4%	2.4%	2.4%
	Model E		1.4%	1.4%	1.4%	1.4%	1.3%	1.4%
% DCIS + IBC overdiagnosis	Model D		2.7%	5.1%	2.5%	4.2%	2.5%	5.0%
	Model E		6.0%	10.5%	5.8%	9.7%	6.7%	8.6%

*DCIS* ductal carcinoma in situ, *IBC* invasive breast cancer, *NA* not available, *df* degree of freedom, *noReg* model without DCIS regression, *wReg* model with DCIS regression

*p values from chi-square tests; mean sojourn times are expressed in number of years

### Mean sojourn times

Models D and E estimated that the MSTs of screen-detectable preclinical DCIS lesions ranged 0.2–7.7 years, but mostly less than 4 years for the different submodels (Table 1). MST estimates varied by submodels and by DCIS progression path (i.e., progress to preclinical IBC, clinical DCIS, or regress). Model D’s estimates showed a trend towards a shorter or equal MSTs in submodel 3 (versus submodels 2 and 1). This trend was not obvious in model E. The MSTs of the lesions that progressed to preclinical IBC were shorter when DCIS regression was allowed. This trend was also seen for the lesions that will progress to clinical DCIS in all submodels for model E and in submodel 1 for model D. The MSTs of the lesions that regressed were 1.5 years in all submodels of model D and varied between 0.6 and 4.0 years in model E.

### Proportion of DCIS cases progressing to other states

**Fig. 4:**
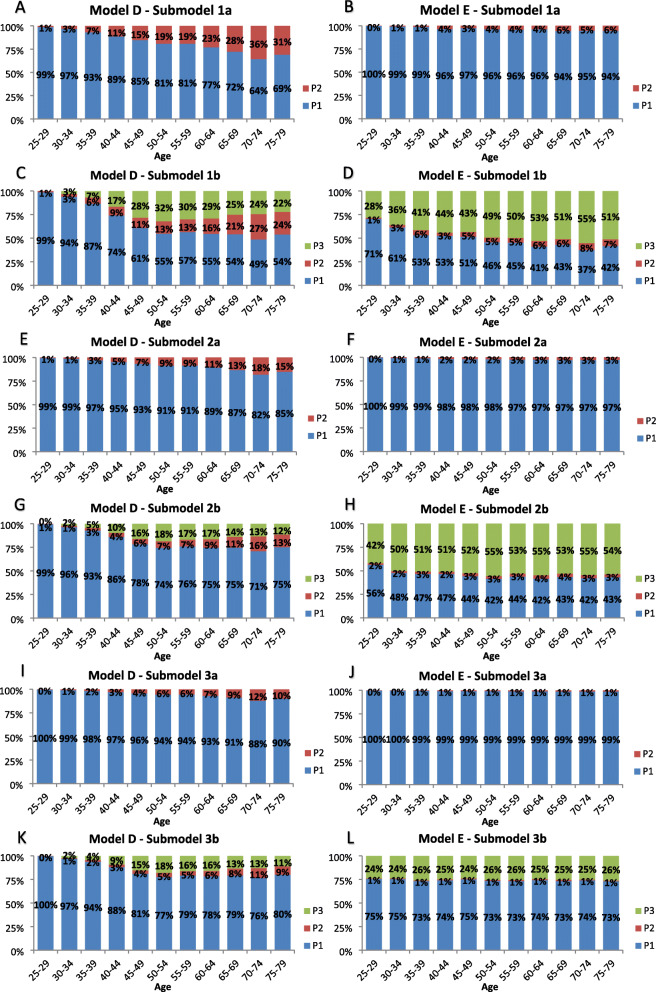
Proportion of DCIS cases progressing to other states. Stacked bar plots showing the proportion of preclinical screen-detectable DCIS progressing to preclinical invasive breast cancer (P1), clinical DCIS (P2), or no breast cancer (P3; regression). P1, P2, and P3 are represented by **blue**, **red,** and **green** bars, respectively. Simulated birth cohort 1930

Figure 4 displays estimated proportions of DCIS lesions in the preclinical DCIS state that progress to other states by age for the 1930 birth cohort in the absence of screening, biopsy, and surgical excision. Assuming no DCIS regression, most submodels showed a large proportion of DCIS progressing to preclinical IBC, ranging from 64 to 100%. For submodels that allowed DCIS regression, this proportion ranged from 36 to 100%. Throughout all submodels, the proportion progressing to clinical DCIS varied from 0 to 36%. In submodels where DCIS regression was allowed, the proportion of DCIS regressing was higher than the proportion progressing to clinical DCIS. Furthermore, Fig. 4 shows that younger women have a larger proportion progressing preclinical IBC compared to older women. Similar proportions of DCIS progression and age-specific trends were observed in all other birth cohorts.

### Overdiagnosis of DCIS and IBC

**Fig. 5:**
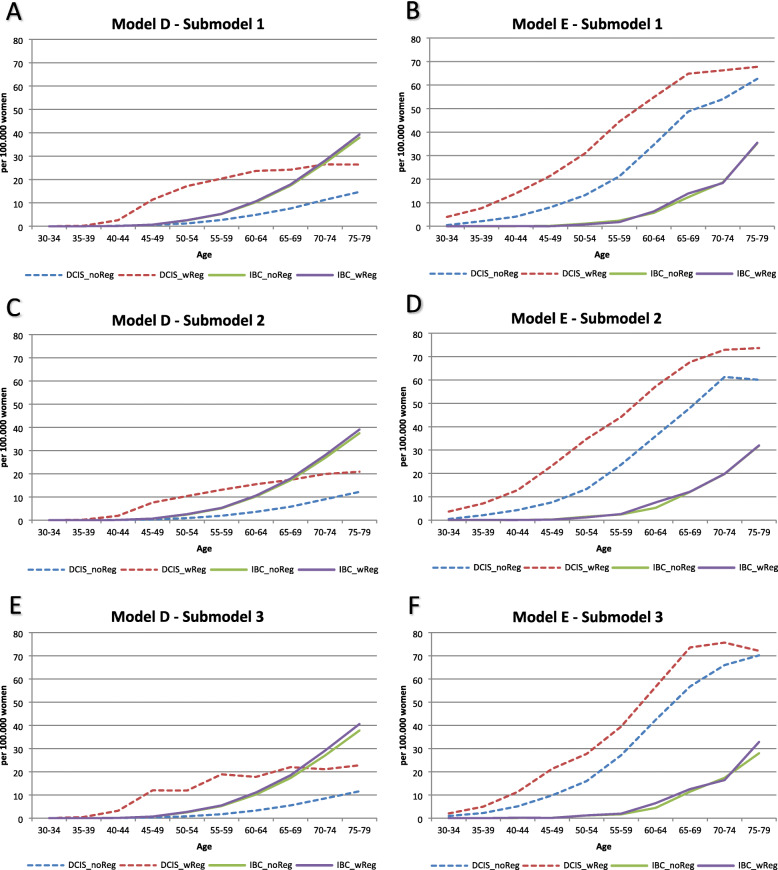
Overdiagnosis by age. Calendar year 2010. Each graph includes overdiagnosis for DCIS and IBC. DCIS, ductal carcinoma in situ; IBC, invasive breast cancer; noReg, submodel without DCIS regression; wReg, with DCIS regression

In both models, the level of DCIS overdiagnosis increased as a function of age and calendar year as mammogram use was more widely disseminated. Both models found a higher overdiagnosis rate when DCIS regression was assumed. For example, model D estimated DCIS overdiagnosis to be 3.1–4.8% across submodels without DCIS regression; overdiagnosis increased to 13.3–19.3% when regression was allowed (Table 1). Model E had a similar trend, although the level of DCIS overdiagnosis was higher (35 to 66%). Combining DCIS and IBC, the level of overdiagnosis varied between 2.5 and 10.5%. Furthermore, both models estimated that IBC overdiagnosis increased with age (see Fig. 5).

## Discussion

As the natural history of DCIS is mostly unknown, it is challenging to pinpoint a unique DCIS model. This study investigated different DCIS natural history models and selected six plausible models that could explain DCIS and IBC incidence in the USA. Unlike other attempts to model the natural history of DCIS [18, 19, 38], our extensive work involved two-established modeling groups, several submodels, multiple birth cohorts, and a 40-year time span with the mammogram dissemination patterns observed in the USA [39]. Our modeling work also showed that several different natural history models fit the observed trends, making any firm conclusions about the DCIS natural history based on observation data difficult.

Most submodels in our study indicated that the majority of unexcised screen-detectable preclinical DCIS lesions progress to IBC. This agrees with previous modeling studies that have estimated progression varying from 61–91% [13, 14, 17, 18]. Notably, results from our submodels showed that younger women may have a higher proportion of progression from unexcised screen-detectable DCIS to preclinical IBC. A possible explanation is that young women tend to have a more aggressive type of DCIS which is more likely to progress to IBC [3]. For women older than 50 years, our study found that the proportion of screen-detectable DCIS progressing to preclinical IBC to be between 36 and 99%. Furthermore, our study showed that the proportion of screen-detectable DCIS that could regress varied between 0 and 56%. These results are consistent with previous modeling studies that have estimated the proportion of DCIS regression was between 1 and 37% [13, 14, 40].

Observational studies of women who did not receive definitive surgery after diagnosis with DCIS have found lower rates of progression invasive cancer, ranging from 12 to 54% [4, 6–12]. However, these studies are not directly comparable to modeling results because they do not capture non-screen detected preclinical DCIS. Indeed, only modeling studies can capture progression rates of lesions uninterrupted by biopsy or other treatment. Lower progression rates in observational studies could be due to a number of factors. First, there is a chance of complete removal of the DCIS lesion during biopsy. This disruption of natural history will bias estimates, resulting in a lower estimated proportion of DCIS progressing to IBC and a non-observed sojourn time. In addition, mammography-detected lesions usually contain calcifications, and it remains unclear whether DCIS with and without calcification have the same natural history. Finally, inflammation of the stroma caused by the biopsy might alter the natural course of DCIS [41].

An interesting finding from our study was that across all submodels the MSTs were relatively short, in particular when assuming DCIS regression. This agrees with previous modeling studies estimating MSTs between 0.5 months to 2.6 years under the assumption that IBC progresses through screen-detectable DCIS [13, 17–19]. Similar to other modeling studies, MSTs tend to be shorter for preclinical screen-detectable DCIS progressing to preclinical IBC compared to other health states such as clinical DCIS or going into regression [13, 19]. Although our results show a similar direction as previous studies, all DCIS modeling studies are subject to considerable residual uncertainty of the estimates. Nevertheless, short MSTs for preclinical screen-detectable DCIS progressing to clinical DCIS and IBC could guide discussions on screening intervals in proposing screening guidelines. With regard to treatment, it remains difficult to make suggestive recommendations about treatment for DCIS patients unless a clinical factor and/or molecular signatures can be identified, providing insight into which women can avoid or postpone treatment [42].

The level of overdiagnosis of breast cancer is challenging to quantify, especially for screen-detected DCIS. Overdiagnosis estimates for DCIS varied between models D and E, with model D showing consistently lower level of DCIS overdiagnosis compared to model E and previous studies [13, 19, 43]. This difference is probably due to model D having shorter estimated MSTs in preclinical DCIS, and high progression rates to IBC, leading to lower DCIS overdiagnosis estimates. In contrast to model D where the sojourn time is used as an input, model E estimates the mean sojourn times for every submodel. Model D used the Norwegian breast cancer screening data to estimate the DCIS model parameters, while model E was developed on US data only.

Our study showed that DCIS overdiagnosis varied from 13 to 66% when DCIS regression was allowed and 3 to 35% when assuming no DCIS regression. Yen et al. [13] also estimated that the proportion of the screen-detected DCIS that is non-progressive (not progressing to IBC) varies from 19 to 46% in the prevalence screen and from 3 to 21% in the first subsequent screen. Another study by Seigneurin et al. estimated that 20.3% (95% CI, 3.0–38.9%) of in situ cancer was overdiagnosed, assuming that non-progressive in situ cancer remains in the preclinical phase [44]. With regard to IBC in our study, overdiagnosis was on average between 1.3 and 2.4% regardless of the assumption of DCIS regression for the age group 30–79 years. This falls in the range of 1–10% for overdiagnosed IBC reported in the systematic review conducted by Puliti et al. based on European studies [45]. Despite the variation in estimated DCIS overdiagnosis levels by submodel, the overall level of overdiagnosis of DCIS+IBC was not high, ranging 2.5–10.5%.

Most submodels in this study showed a reasonable fit with SEER data, indicating that different sets of parameters can match observed breast cancer rates. The ability to fit population data with varied parameters highlights the difficulty in providing definitive conclusions on the natural history of DCIS. This difficulty makes the pending results of the currently enrolling active monitoring trials LORD, LORIS, and COMET even more critical [20–22]. Although these trials focus on the disease progression of screen-detected, biopsied low-risk lesions, they will significantly enhance our understanding of overall clinical management of DCIS. Further research should focus on the heterogeneity of DCIS in various age groups as younger women may tend to have a more aggressive type of DCIS. Also, future modeling work by DCIS grade, molecular subtype, and inherent factors such as family history of breast cancer will provide more specific information on MSTs and progression of the disease that will contribute to guiding women with specific features on screening and treatment.

## Conclusion

Our study suggested that the majority of unexcised screen-detectable preclinical DCIS lesions progress to IBC and that the MSTs are relatively short. Furthermore, our modeling work also showed that several different natural history models fit the observed trends, making any firm conclusions about the DCIS natural history based on observation data difficult. Due to the heterogeneity of DCIS, more research is needed to understand the progression of DCIS by grades, molecular subtypes, and certain inherent factors such as family history of breast cancer.

## Supplementary information

**Additional file 1.** Model D description.

**Additional file 2.** Model E description.

## Data Availability

The datasets used and/or analyzed during the current study are available from the corresponding author on reasonable request.

## Abbreviations

DCIS: Ductal carcinoma in situ
IBC: Invasive breast cancer
MST: Mean sojourn time
SEER: Surveillance, Epidemiology and End Results
CISNET: Cancer Intervention and Surveillance Modeling Network
Model D: Model developed by Dana-Farber Cancer Institute
Model E: Model developed by Erasmus MC, University Medical Center Rotterdam
USPSTF: United States Preventive Services Task Force
BCSC: Breast Cancer Surveillance Consortium
