# Supplementary material for: Modeling the natural history of ductal carcinoma in situ based on population data
Source: Breast Cancer Res. 2020 May 27;22:53. doi: 10.1186/s13058-020-01287-6 (PMC7251719; doi:10.1186/s13058-020-01287-6)
Supplement: Supplementary file 1 — Additional file 1. Model D description. [file 13058_2020_1287_MOESM1_ESM.docx]

**Supplementary A**

**Model D**

**Background:** Model D developed a stochastic model that characterizes DCIS progression using health states and transitions among the health states (Figure 1). Applying the DCIS stochastic model, model D derived the probability expressions for DCIS case-finding processes in mammography screening and formulated the corresponding likelihood functions. In the absence of an adequate data set to model DCIS natural history, model D constructed the data from the Norwegian Breast Cancer Screening Program (NBCSP) to resemble datasets from screening trials by screening round and mode of detection. Using the NBCSP data, the maximum likelihood estimates (MLE) for the key DCIS natural history parameters were obtained (Table 1).

**Formulation and Notation:** Health states are defined as: (1) disease-free (S_0_), (2) early stage DCIS not detectable by any screening modality (S_du_), (3) preclinical DCIS detectable by screening (S_dp_), (4) clinical DCIS with symptoms (S_dc_), (5) preclinical invasive breast cancer detectable by screening (S_p_), (6) clinical invasive breast cancer with symptoms (S_c_), and (7) breast cancer death (S_d_). For the transition probabilities between health states, we define *W_0_(t)∆t* as the transition probability of S_0_ → S_du_ during an age interval *(t, t+ ∆t)*. Other transition probabilities are *W(t)* for S_du_ → S_dp_, *I_1_(t)* for S_dp_ → S_p_, *I_2_(t)* for S_dp_ → S_dc_, *I_3_(t)* for S_dp_ → S_du_, *W_i_(t)* for S_du_ → S_p_, *I_IBC_(t)* for S_p_ → S_c_ and *D(t)* for S_c_ → S_d_.

The model envisions that screen-detectable preclinical DCIS (S_dp_) can: (1) progress to preclinical invasive breast cancer that can be screen-detectable (S_p_), (2) progress to a clinical DCIS state (S_dc_), or (3) regress to no cancer state. Transition probabilities for the three paths from S_dp_ are *I_1_(t), I_2_(t)* and *I_3_(t)*, respectively. The overall transition probability of entering S_dp_, *W(t)*, can be decomposed into three parts: *W(t) = W_1_(t) + W_2_(t) + W_3_(t)*, where *W_1_(t), W_2_(t)* and *W_3_(t)* correspond to transition probabilities that will transit out of S_dp_ as specified in paths (1), (2) and (3), respectively. An Exponential probability density function (pdf) of sojourn time in S_dp_ was considered with: $q_{g}\left( t \right)=\frac{1}{m_{g}(z)}\exp\left( -\frac{t}{m_{g}\left( z \right)} \right), t>0$where *z* represents age, *m_g_(z)* is mean sojourn time at age *z* where g=1, 2, 3 for the three paths out of S_dp_ as described above. Transition probabilities *I_2_(t)* and *I_IBC_(t)* can be directly calculated from the age-specific incidence of clinical DCIS and invasive breast cancer (IBC) in the absence of screening, biopsy or excision. For the US model, *I_2_(t)* and *I_IBC_(t)* were estimated from DCIS and IBC incidence generated from the US Age-Period-Cohort (APC) model. All other transition probabilities are unobservable, but can be estimated using the proposed model.

**Parameter Estimation:** Model D formulated the probability of screening detection or interval diagnosis of DCIS cases in screening programs where mammography is administered repeatedly. Consider a screening program where a mammography is repeated at ages: *t_0_ < t_1_ < t_2_ < … < t_n-1_*_._ The r^th^ interval is defined as (*t_r-1_, t_r_*]. Individuals in the program can be detected at the screening examinations or diagnosed as interval cases. $D_{r}$ denotes the probability of being screen-detected at *t_r_* and $I_{{int}_{r}}$ denotes the probability of interval-diagnosis between *t_r-1_* and *t_r_* for r $\geq$ 1. Both $D_{r}$ and $I_{{int}_{r}}$ are a function of transition probabilities *W_g_(t)*, sojourn time distributions in S_dp,_ *q_g_(t)*, and mammogram sensitivity *β(t)* for *g=1,2.3*. For the simplicity of estimation, first mean sojourn time in S_dp_*,* *m_g_(z)=m* was assumed to be the same for *g*=1,2,3. These formulations were incorporated to build a likelihood function:

$$L\left( \boldsymbol{m,W} \right)=\prod_{r=0}^{n-1} L_{r}\left( \boldsymbol{m,W} \right)= D_{r}^{N_{scr,r}}I_{{int}_{r}}^{N_{int,r}}\left( 1-D_{r}-I_{{int}_{r}} \right)^{N_{tol,r}-N_{scr,r}-N_{int,r}}$$

where *N_scr,r_* denote the number of DCIS screen-detected individuals at *t_r_*, *N_int,r_* the number of DCIS interval-diagnosed individuals between *t_r_* and *t_r+1_*, and *N_tol,r_* the total number of individuals screened at *t_r_.* In obtaining MLEs from the likelihood function, transition probabilities *W_2_(t)* and *W_1_(t)* were first estimated with an initial mean sojourn time (m), *I_2_(t)* and *I_1_(t*), and updated in the likelihood function. A convergence of MLEs was achieved for parameters in submodel 1 with the assumption of *m=m_1_=m_2_=m_3_,* but not in submodels 2 and 3 (Figure 1). For submodels 2 and 3, *m_2_(z)* was estimated using *I_2_(t),* and various combinations of (*m_1_(z), m_3_(z))* were evaluated. The values of (*m_1_(z), m_3_(z)*) that could generate the DCIS incidence in an independent data source (Norway Survey Data of DCIS incidence based on the time from the last mammography screening) were selected (Table 1).
