# Supplementary material for: Modeling the natural history of ductal carcinoma in situ based on population data
Source: Breast Cancer Res. 2020 May 27;22:53. doi: 10.1186/s13058-020-01287-6 (PMC7251719; doi:10.1186/s13058-020-01287-6)
Supplement: Supplementary file 2 — Additional file 2. Model E description. [file 13058_2020_1287_MOESM2_ESM.docx]

**Supplementary B**

**Model E**

**Background:** Model E, also known as Miscan-Fadia, is a discrete-event driven micro-simulation model (1). Life histories of each individual woman are simulated, which consist of various events such as birth, onset of the DCIS and IBC tumor, clinical diagnosis and death from breast cancer or other causes. The natural history parameter for invasive breast cancer have been estimated based on the Swedish Two County study (1, 2).

**DCIS submodel:** The DCIS submodel in MISCAN-Fadia is state-based and includes the following health states: preclinical undetectable DCIS, preclinical screen-detectable DCIS, clinical DCIS and regressive DCIS (1, 3). From the normal state, tissue can progress to preclinical undetectable DCIS. The preclinical undetectable DCIS can either become screen-detectable or preclinical IBC, which was initially assumed to be equally likely. When the state of preclinical screen-detectable DCIS has been reached, it can progress to clinical DCIS, preclinical IBC or go into regression. Transition rates between the health states were estimated based on data from Surveillance, Epidemiology, and End Results Program (SEER). The model assumes that the sojourn time for each DCIS state is exponentially distributed. Furthermore, the model also assumes that the BC survival of women with DCIS (both clinically detected and screen-detected DCIS) is 100%. The onset of DCIS is dependent on the age and birth cohort of a woman. The age factor between 20 and 90 are multiplied by transition rates. Therefore, P1 will be relatively higher for young women than for older women. When DCIS turns invasive, it does not disappear. A woman can have both DCIS and IBC; however only one of the two lesions can be detected depending on the visibility of the lesion whereby the visible IBC will always be detected first if both lesions are visible.

**Model calibration:** Using the MISCAN-Fadia model, several key parameters for each submodel in this study were calibrated to U.S. SEER data (DCIS and IBC incidence) between 1975 and 2015 for the age group 30-79 year. The estimated parameters were age-dependent onset rates and different transition rates between the aforementioned health states. Each submodel includes two assumption that is specific for that particular submodel. Firstly, a submodel assumes a proportion, 30%, 50% or 80%, that represents the progression from preclinical undetectable DCIS to preclinical screen-detectable DCIS. Secondly, a submodel can either assume the existence or nonexistence of regressive DCIS. Through calibration, each submodel has an optimized set of parameter values that matches best with the SEER incidence data given the two specified assumptions.

**Key parameters:** Two important parameters in the submodels influence the incidence level of DCIS. First, the age dependent onset parameters determines the number of women who will develop DCIS at a certain age. Second, the proportion (e.g. 30%, 50%, or 80%) that transits from preclinical undetectable DCIS to preclinical screen-detectable DCIS also affects the number of DCIS.

With regard to the transition from preclinical screen-detectable DCIS to other health states, each progression/regression path is represented by a parameter P1, P2 or P3 (Figure 1). The ratio between these three parameters determines the proportion transitioning from preclinical screen-detectable DCIS to the next health state.

**Early detection of DCIS in Mammography Screening:** When DCIS is in the preclinical screen-detectable state, it can be detected by mammography screening. The detection of DCIS depends on the sensitivity of mammography . In this study, we used the following sensitivities 0.45, 0.55, 0.7, 0.85 for the calendar years 1975, 1985, 2000, and 2010, respectively(4). These sensitivities apply to all ages.

**Mean sojourn times:** For each path (P1, P2 or P3), the model estimates for every woman the sojourn times. There is a competing risk among the three path ways, meaning that for each woman the path which has the shortest sojourn time, will be included into the calculation of the mean sojourn time.

1. Tan SYGL, Van Oortmarssen GJ, De Koning HJ, Boer R, Habbema JDF. Chapter 9: the MISCAN-Fadia continuous tumor growth model for breast cancer. JNCI Monographs. 2006;2006(36):56-65.

2. Tabar L, Fagerberg G, Duffy SW, Day NE, Gad A, Gröntoft O. Update of the Swedish two-county program of mammographic screening for breast cancer. Radiologic Clinics of North America. 1992;30(1):187-210.

3. van Ravesteyn NT, van den Broek JJ, Li X, Weedon-Fekjær H, Schechter CB, Alagoz O, et al. Modeling ductal carcinoma in situ (DCIS): An overview of CISNET model approaches. Med Decis Making. 2018;38(1_suppl):126S-39S.

4. Lee S, Zelen M. Chapter 11: a stochastic model for predicting the mortality of breast cancer. JNCI Monographs. 2006;2006(36):79-86.
